# Registered Reports offer recognition for rigour

**DOI:** 10.1038/s41467-020-17294-2

**Published:** 2020-07-09

## Abstract

*Nature Communications* are pleased to announce that we are now considering Registered Reports for publication in the fields of cognitive neuroscience, human behaviour and psychology, as well as epidemiology.

In research, findings from one study are often the starting point of another. Thus it is essential that investigators have high confidence that results were obtained following the best available methods and are reproducible.

At *Nature Communications*, we have already taken several steps to increase reproducibility in research. We have adopted measures to standardise the reporting of methods ahead of peer review. To increase transparency in our editorial processes, we give authors the option to publish alongside their article the reports from the reviewers and their own responses, thereby providing insights into the scientific discussion that took place before publication. We also offer reviewers the option to be recognised by name for their work in the peer-review process. We are now expanding our measures to improve reproducibility in research by considering Registered Reports for publication.

Registered Reports are a type of article where a study proposal is peer reviewed before the research is undertaken. Reviewers focus their comments on the validity of the research questions and the thoroughness and quality of the study protocol. Registered Reports that meet high scientific standards are then editorially accepted by the journal before the research is carried out and results are known. Once the study is performed and the final article submitted, reviewers are contacted to ensure the original protocol was followed but are not asked to comment on the results nor to request additional experiments. Introduced in 2013, the Registered Report format is now offered at more than 240 journals including our sister journal, *Nature Human Behaviour*.

By shifting the emphasis to the quality of the study plan as a condition for publication, the Registered Report format reduces publication bias and offers opportunities to improve the reproducibility of research. The format also provides an avenue for publishing negative results for research questions of high importance.

As a journal, we strive to foster early recognition of contributions made by researchers by supporting preprint deposition of research articles. Once the Registered Report is in principle accepted for publication ahead of the investigation, authors will have to pre-register their study plan in a dedicated registry. They can also receive early credit for their research if they choose to publicly disclose it at this stage. Further details and guidelines for authors and reviewers appear on our website.

We are currently open to Registered Reports submissions in the fields of cognitive neuroscience, human behaviour, psychology, and epidemiology. We are particularly interested in manuscripts that propose studies to distinguish between competing hypotheses and replication studies of influential work.

Given their strong quantitative component, the quality of epidemiological studies is often determined by the choice of populations and the analysis plan. In this context, we welcome Registered Reports planning to look at transmission of infectious diseases and to monitor population trends for other health-related issues.

We also believe that the benefits offered by this article format are highly relevant for coronavirus disease 2019 (COVID-19)-related research. We are thus pleased to join an initiative launched by Chris Chambers at *Royal Society Open Science* to expedite review of Registered Reports for COVID-19. As part of this initiative, 700 researchers from a wide range of disciplines pledged to provide subject-specific guidance in a very short time frame. With their help, we will aim to peer review initial study plans within 10 days of submissions and waive article processing charges for COVID-19 Registered Reports receiving an in-principle acceptance decision before the end of 2020.

In this area, we are particularly interested in study plans that have strong policy implications. We encourage submissions that propose to analyse behavioural and psychological impacts of the pandemic, such as those aiming to test behavioural interventions for reducing both the spread of the virus and of COVID-19-related misinformation. We are also interested in proposals for observational studies that estimate severe acute respiratory syndrome coronavirus 2 infection rates in different populations, establish risk factors and effects of interventions, and determine interactions with other viruses.

Offering in-principle acceptance based on the review of the study protocol fosters more rigorous practices in research by focusing the condition for publication on the strength of the methodologies.

Offering in-principle acceptance based on the review of the study protocol fosters more rigorous practices in research by focusing the condition for publication on the strength of the methodologies. It has already been shown to reduce publication bias for positive results. The approach also supports early career researchers by offering them the assurance of a publication at the end of their project. In the future, we intend to open the format to all communities served by the journal. We are interested to know how this article format can specifically benefit studies in your area. You can send any comments to naturecommunications@nature.com

